# Real-world experience with molnupiravir during the period of SARS-CoV-2 Omicron variant dominance

**DOI:** 10.1007/s43440-022-00408-6

**Published:** 2022-08-24

**Authors:** Robert Flisiak, Dorota Zarębska-Michaluk, Magdalena Rogalska, Justyna Anna Kryńska, Justyna Kowalska, Ewa Dutkiewicz, Krystyna Dobrowolska, Jerzy Jaroszewicz, Anna Moniuszko-Malinowska, Marta Rorat, Regina Podlasin, Olga Tronina, Piotr Rzymski

**Affiliations:** 1 Department of Infectious Diseases and Hepatology, Medical University of Białystok, 15-540, ul. Żurawia 14, Białystok, Poland; 2 Department of Infectious Diseases, Jan Kochanowski University, Kielce, Poland; 3 Provincial Hospital, Kielce, Poland; 4 Department of Adults’ Infectious Diseases, Medical University of Warsaw, Warsaw, Poland; 5 ZOZ, Busko Zdroj, Poland; 6 Jan Kochanowski University, Collegium Medicum, Kielce, Poland; 7 Department of Infectious Diseases and Hepatology, Medical University of Silesia, Katowice, Poland; 8 Department of Infectious Diseases and Neuroinfections, Medical University of Białystok, Białystok, Poland; 9 Department of Forensic Medicine, Wrocław Medical University, Wrocław, Poland; 10 IV-th Department, Hospital for Infectious Diseases, Warsaw, Poland; 11 Department of Transplantation Medicine, Nephrology, and Internal Diseases, Medical University of Warsaw, Warsaw, Poland; 12 Department of Environmental Medicine, Poznan University of Medical Sciences, Poznan, Poland

**Keywords:** Molnupiravir, COVID-19, SARS-CoV-2, Omicron, Treatment, Real-world experience

## Abstract

**Background:**

The real-world effectiveness of molnupiravir (MOL) during the dominance of Omicron SARS-CoV-2 lineage is urgently needed since the available data relate to the period of circulation of other viral variants. Therefore, this study assessed the efficacy of MOL in patients hospitalized for COVID-19 in a real-world clinical practice during the wave of Omicron infections.

**Methods:**

Among 11,822 patients hospitalized after 1 March 2020 and included in the SARSTer national database, 590 were treated between 1 January and 30 April 2022, a period of dominance of the Omicron SARS-CoV-2 variant. MOL was administered to 203 patients, whereas 387 did not receive any antiviral regimen. Both groups were similar in terms of sex, BMI and age allowing for direct comparisons.

**Results:**

Patients who did not receive antiviral therapy significantly more often required the use of Dexamethasone and Baricitinib. Treatment with MOL resulted in a statistically significant reduction in mortality during the 28-day follow-up (9.9 vs. 16.3%), which was particularly evident in the population of patients over 80 years of age treated in the first 5 days of the disease (14.6 vs. 35.2%). MOL therapy did not affect the frequency of the need for mechanical ventilation, but patients treated with MOL required oxygen supplementation less frequently than those without antivirals (31.7 vs. 49.2%). The time of hospitalization did not differ between groups.

**Conclusions:**

The use of molnupiravir in patients hospitalized for COVID-19 during the dominance of Omicron variant reduced mortality. This effect is particularly evident in patients over 80 years of age.

## Introduction

The first infections of the novel coronavirus 2019 (2019-nCov), later named severe acute respiratory syndrome coronavirus 2 (SARS-CoV-2), a causative agent of the coronavirus disease 2019 (COVID-19), were documented in China in December 2019. The rapid spread of the disease and an increasing number of infections prompted World Health Organization (WHO) to declare COVID-19 a pandemic as early as March 2020. The clinical spectrum of respiratory infections ranges from mild to critical, with several percent of patients dying from respiratory failure, shock, and multiple organ failure. Due to such consequences, the number of cases quickly made the disease one of the major sources of morbidity and mortality worldwide. Virus genetic variability posed an additional problem, as successive variants differed not only in their infectivity and pathogenicity but also in the effectiveness of the drugs used against them. This became most apparent with the currently dominant Omicron variant [1, 2].

Since the beginning of the pandemic, an urgent need for effective forms of therapy has arisen to reduce this global health burden. Due to the lack of time needed to create a new drug, the researchers’ attention has focused on using existing drugs for a new indication [3]. Thus, MOL, an antiviral drug originally developed to treat patients with Venezuelan equine encephalitis virus infection and later, in the pre-pandemic period, entered preclinical studies with influenza, has come into the spectrum of attention [3–6]. MOL, a polymerase inhibitor prodrug that acts as a synthetic nucleoside, is administered orally, an advantage over the previously available intravenous, antiviral drug, remdesivir, used to treat COVID-19 and facilitates therapy in the out-of-hospital setting [7]. It was the phase 2/3 MOVe-OUT clinical trial in non-hospitalized patients with SARS-CoV-2 infection that was the basis for the Food and Drug Administration (FDA) December 2021 approval of MOL for emergency use in the treatment of mild-to-moderate COVID-19 virus infection in adults at high risk of progression to severe disease [8]. No clinical benefit was seen in the MOVe-IN clinical trial evaluating MOL in hospitalized patients [9]. This study, however, was conducted in the period of dominance of the earlier SARS-CoV-2 variants and does not necessarily reflect the current situation related to the dominance of the Omicron variant.

The purpose of this analysis was to assess the efficacy of MOL in patients, particularly the elderly, hospitalized for COVID-19 in a real-world clinical practice during the period of the Omicron variant dominance.

## Patients and methods

Patients included in the analysis originated from the SARSTer national database, which included 11,822 patients hospitalized between 1 March 2020 and 30 April 2022 in 30 Polish centers. This ongoing project, supported by the Polish Association of Epidemiologists and Infectiologists (PTEiLChZ), is a national real-world experience study assessing treatment in patients with COVID-19. The study was conducted according to the guidelines of the Declaration of Helsinki, and approved by the Ethics Committee of the Medical University of Białystok (29 October 2020, number APK.002.303.2020). All the patients were diagnosed with COVID-19 based on positive results of the real-time reverse transcriptase-polymerase chain reaction (RT-PCR) or using an antigen test from the nasopharyngeal swab specimen. The decision about the treatment regimen was taken entirely by the treating physician concerning current knowledge and recommendations of the PTEiLChZ [10, 11]. The present study included 590 adult patients from 13 centers, hospitalized between 1 January and 30 April 2022, which is considered the period of dominance of the Omicron SARS-CoV-2 variant in Poland [12]. Patients receiving other antiviral drugs, especially remdesivir, were not included. The total number of patients hospitalized in these 13 centers during this period was 801. Molnupiravir was administered orally twice daily at 800 mg for 5 days to 203 patients according to PTEiLChZ recommendations, whereas 387 did not receive any antiviral regimen [10, 11]. Data were entered retrospectively and submitted online by a web-based platform operated by Tiba sp. z o.o. Baseline data included age, gender, body mass index (BMI), coexisting conditions, use of other COVID-19 medications, clinical and laboratory measures including C-reactive protein (CRP), procalcitonin, white blood cells (WBC), platelets, interleukin 6 (IL-6), and d-dimers. The information on vaccination status was not available for patients considered in this study. The end-points and how to analyze the data was established before starting the data evaluation. Treatment effectiveness end-points were 28 days mortality rate and the need for mechanical ventilation. Moreover, clinical improvement was analyzed with ordinal scale categories at consecutive time points on day 7, 14, 21, or 28 depending on baseline oxygen saturation (SpO_2_), patient's age and molnupiravir administration within 5 days of symptom onset. This scale is based on WHO recommendations modified to fit the specificity of the national health care system as applied previously [13, 14]. The ordinal scale was scored as follows: (1) unhospitalized, no activity restrictions; (2) unhospitalized, with limited activity; (3) hospitalized, does not require oxygen supplementation or medical care; (4) hospitalized, requiring no oxygen supplementation, but requiring medical care; 5) hospitalized, requiring normal oxygen supplementation; (6) hospitalized, on non-invasive ventilation with high-flow oxygen equipment; (7) hospitalized, on invasive mechanical ventilation or extracorporeal membrane oxygenation (ECMO); (8) death.

### Statistical analyses

The results were expressed as mean ± standard deviation (SD) or *n* (%). All statistical calculations were performed with Statistica v. 13.3 (StatSoft, USA). Since the data distribution did not meet the Gaussian assumption, non-parametric methods (Mann–Whitney U test) were applied to assess the difference between MOL and NO AVT groups. Differences in frequencies in parameters given as nominal categorical variables were evaluated with Pearson's χ^2^ test. A p-value < 0.05 was considered statistically significant.

## Results

As shown in Table 1, the study groups were similar in terms of sex, BMI and age, with as many as 73% of patients treated with MOL and 71% of the untreated group over 60 years of age. Except for platelet count, there were no significant differences between the groups of patients in the baseline SpO_2_ and values of major laboratory indicators used in monitoring the course of COVID-19 (Table 1). Patients who did not receive antiviral therapy significantly more often required the use of immunomodulating drugs during 28 days follow-up period, especially dexamethasone and baricitinib (Table 1).

**Table 1:** Clinical characteristics of all adult patients hospitalized between 1-01-2022 and 30-04-2022 receiving molnupiravir (MOL) or no antiviral therapy (NO AVT)

	MOL *n* = 203	NO AVT *n* = 387	*p*
Females/males, *n* (%)	113/90 (56/44)	211/176 (55/45)	Χ^2^ = 0.07 *p = *0.791
BMI, mean ± SD	26.5 ± 5.2	27.2 ± 5.2	*U* = 5787 *p = *0.149
Age, mean ± SD	67.4 ± 17.9	67.4 ± 19.4	*U* = 8324 *p = *0.504
Age, years, *n* (%)			
< 20	1 (0)	1 (0)	*Χ*^2^ = 0.22 *p = *0.641
20–40	21 (10)	51 (13)	*Χ*^2^ = 1.0 *p = *0.318
40–60	34 (17)	62 (16)	*Χ*^2^ = 0.05 *p = *0.820
60–80	91 (45)	153 (40)	*Χ*^2^ = 1.5 *p = *0.215
> 80	56 (28)	120 (31)	*Χ*^2^ = 0.75 *p = *0.388
Baseline measures			
SpO_2_ < 95%, *n* (%)	107 (53)	228 (59)	*Χ*^2^ = 2.1 *p = *0.149
CRP, mg/l, mean ± SD	52 ± 60	70 ± 84	*U* = 33,382 *p = *0.155
Procalcitonin, ng/ml, mean ± SD	1.6 ± 8.2	1.8 ± 9.2	*U* = 31,535 *p = *0.650
WBC, /μl, mean ± SD	8027 ± 9147	7855 ± 4663	*U* = 35,964 *p = *0.112
PLT, /μl, mean ± SD	199,512 ± 94,489	215,395 ± 90,603	***U* = 34,769** ***p = *0.03**
IL-6, pg/ml, mean ± SD	97 ± 316	99 ± 264	*U* = 23,568 *p = *0.349
d-dimers, ng/ml, mean ± SD	1914 ± 3029	2075 ± 3570	*U* = 37,337 *p = *0.738
Additional medication for COVID-19, n (%)			
Low-molecular heparyn	113 (56)	221 (57)	*Χ*^2^ = 0.11 *p = *0.737
Dexamethasone	43 (21)	133 (34)	***Χ***^**2**^** = 11.1** ***p = *0.0008**
Tocilizumab	12 (6)	38 (10)	*Χ*^2^ = 2.6 *p = *0.105
Baricitinib	0	24 (6)	***Χ***^**2**^** = 13.1** ***p = *0.0003**

Statistical significance is indicated with bold italic

**Fig. 1:**
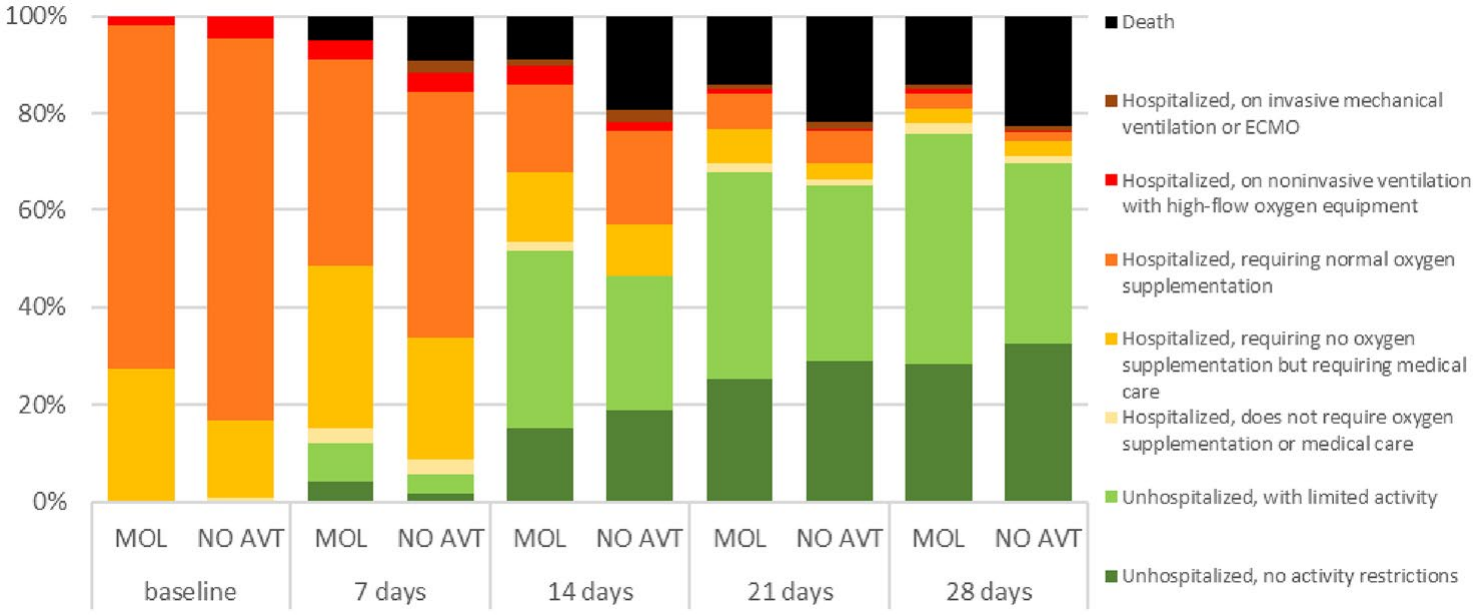
Ordinal scale categories at consecutive time points in all patients with SpO_2_ ≤ 95% treated with molnupiravir (MOL) within 5 days of symptom onset or no antiviral treatment (NO AVT); the bar sections represent the proportions of the ordinal scale categories

**Fig. 2:**
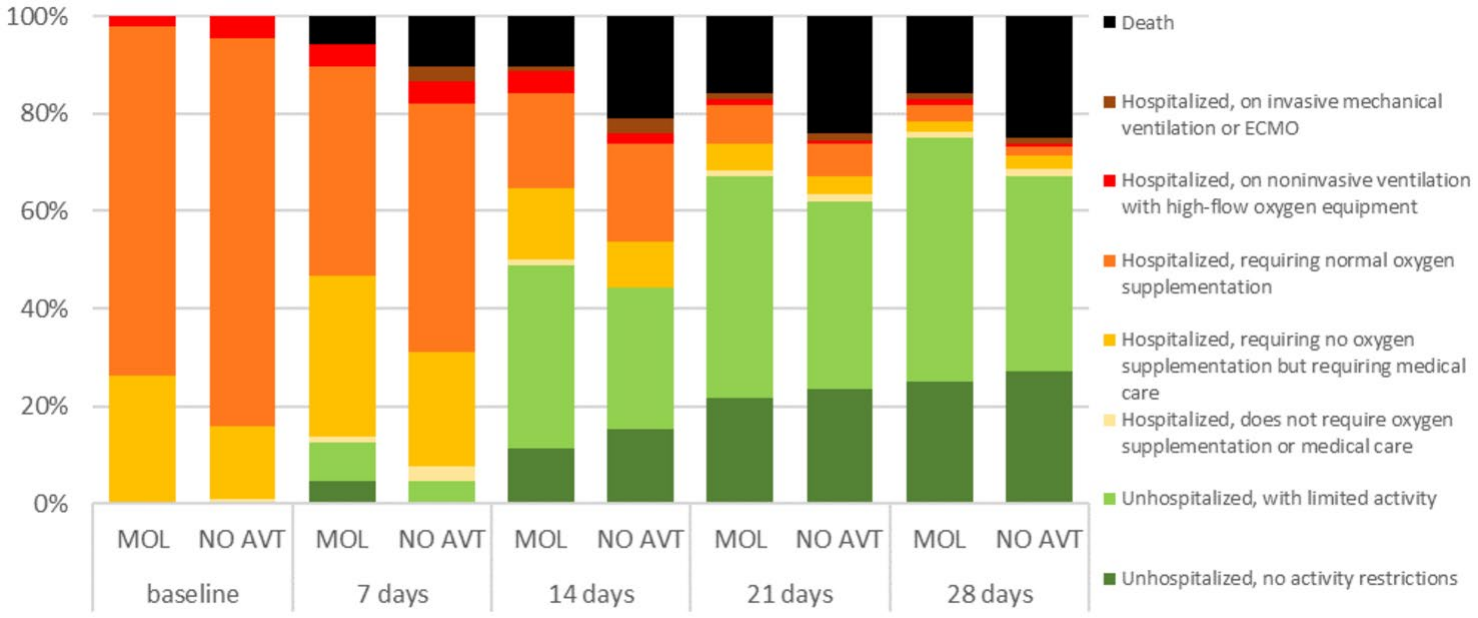
Ordinal scale categories at consecutive time points in patients over 60 years of age with SpO_2_ ≤ 95% treated with molnupiravir (MOL) within 5 days of symptom onset or no antiviral treatment (NO AVT); the bar sections represent the proportions of the ordinal scale categories

**Fig. 3:**
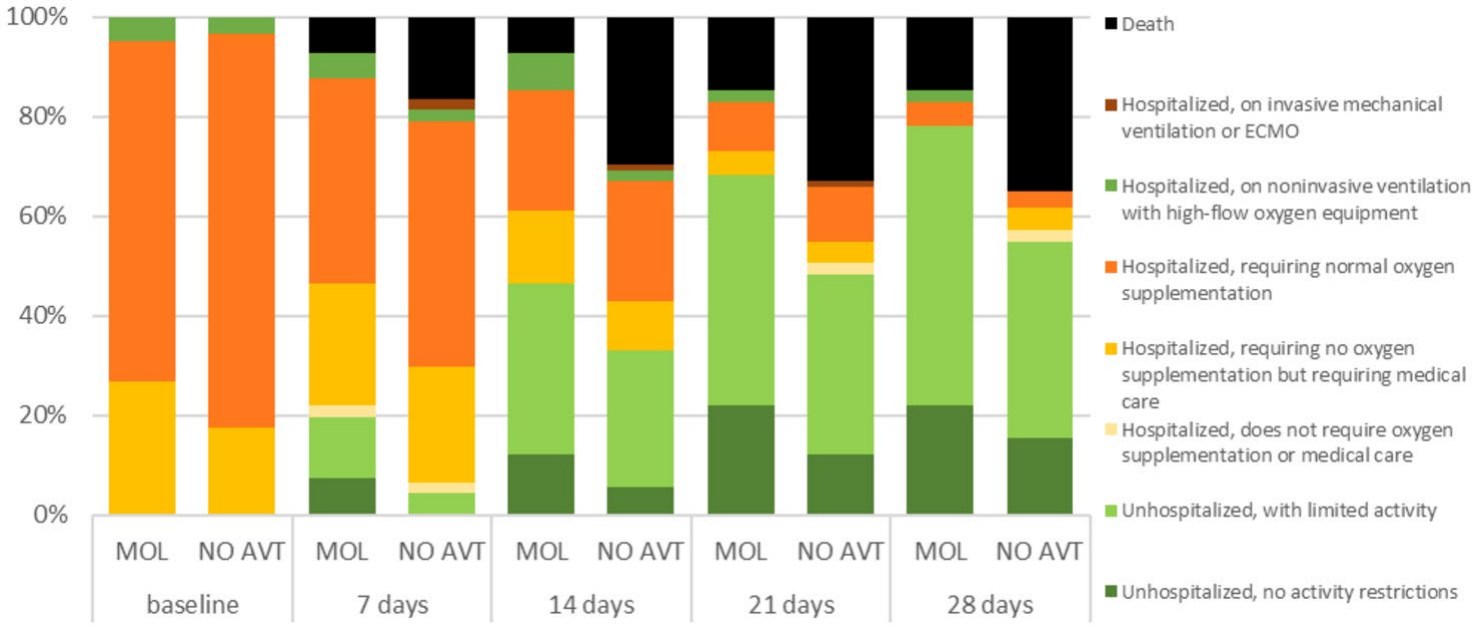
Ordinal scale categories at consecutive time points in patients over 80 years of age with SpO_2_ ≤ 95% treated with molnupiravir (MOL) within 5 days of symptom onset or no antiviral treatment (NO AVT); the bar sections represent the proportions of the ordinal scale categories

A group of patients who received MOL within the first 5 days of the disease was characterized from the 7th day of disease by the tendency for a lower percentage of deaths (5.1 vs. 9.2%, *p = *0.05), the need for oxygen therapy (42.4 vs. 50.4, *p = *0.06) and a significantly higher percentage of hospital discharge (12.1 vs. 5.7%, *p = *0.005) (Fig. 1). The differences in the death rates between groups (5.7 vs. 10.3%, *p = *0.01) at day 7 increased in patients over 60 years of age (Fig. 2), and especially in those over 80 (7.3 vs. 16.5%, *p = *0.03), (Fig. 3). As shown in Table 2, treatment with MOL resulted in a statistically significant reduction in mortality during the 28-day follow-up. This was particularly evident in the population of patients over 80 years of age who took the drug in the first 5 days of the disease. However, antiviral therapy did not affect the frequency of the need for mechanical ventilation (Table 2).

**Table 2:** Twenty-eight days mortality and the need for mechanical ventilation in patients treated with molnupiravir (MOL) or without any antiviral treatment (NO AVT) analyzed in all patients, only those with oxygen saturation ≤ 95%, including administration of molnupiravir within 5 days of symptom onset as well as aged over 60 or 80 years

Patients	Mortality			Mechanical ventilation		
subpopulations	MOL *n*/*N* (%)	NO AVT *n*/*N* (%)	*P*	MOL *n*/*N* (%)	NO AVT n/N (%)	*p*
All patients	20/203 (9.9)	63/387 (16.3)	***Χ***^**2**^** = 4.6** ***p = *0.03**	7/203 (3.5)	14/387 (3.6)	*Χ*^2^ = 0.01 *p = *0.916
SpO_2_ ≤ 95%	17/107 (15.9)	52/228 (22.8)	*Χ*^2^ = 2.1 *p = *0.114	5/107 (4.7)	11/228 (4.8)	*Χ*^2^ = 0.009 *p = *0.952
SpO_2_ ≤ 95%, 0–5 days,	14/99 (14.1)	52/228 (22.8)	*Χ*^2^ = 3.2 *p = *0.073	3/99 (3.0)	11/228 (4.8)	*Χ*^2^ = 0.54 *p = *0.461
SpO_2_ ≤ 95%, 0–5 days, > 60 years	14/88 (15.9)	49/195 (25.1)	*Χ*^2^ = 3.1 *p = *0.08	3/88 (3.4)	11/195 (5.6)	*Χ*^2^ = 0.64 *p = *0.423
SpO_2_ ≤ 95%, 0–5 days, > 80 years	6/41 (14.6)	32/91 (35.2)	***Χ***^**2**^** = 5.8** ***p = *0.016**	0/41 (0)	3/91 (3.3)	*Χ*^2^ = 1.4 *p = *0.239

*N* number of patients included in particular subpopulation, *n* number of patients who died or required mechanical ventilation

The time of hospitalization did not differ between patients treated with MOL and without any antiviral treatment (mean ± SD 11.6 ± 7.9 vs. 11.5 ± 9.3 days; *p = *0.965), and this lack of difference was also observed in the subset of patients aged > 60 years (14.1 ± 9.1 vs. 13.9 ± 9.3 days; *p = *0.679) and 80 years (14.2 ± 9.6 vs. 14.01 ± 11.4 days; *p = *0.855). All patients treated with MOL during the whole 28-days follow-up period required oxygen supplementation less frequently than those not treated with any antivirals (31.7 vs. 49.2%, *p = *0.00005). This difference was also evident in the subset of patients aged > 60 years (56.3 vs. 73.8%, *p = *0.003) but not in the subset of patients > 80 years (62.5 vs 69.3%, *p = *0.449).

## Discussion

Although the MOVe-IN clinical trial evaluating the use of MOL in a population of hospitalized patients was discontinued due to a lack of expected benefits, data from both the MOVe-OUT and MOVe-IN studies demonstrated that MOL appears to inhibit replication of the virus and is the most effective when therapy is started early in the disease course in patients with mild-to-moderate COVID-19 [8, 9]. The consequence of discontinuing the clinical trial in hospitalized patients was that the FDA did not issue an emergency use authorization of MOL in this population, and there was a paucity of reports of treatment efficacy in such patients in the real-world population, making our analysis unique. To date, the only study from routine clinical practice on the use of MOL in hospitalized patients has been published as a preprint and presents the experience of a center in Hong Kong, confirming the efficacy of oral antivirals, including MOL used in 2116 patients, in reducing mortality and risk of disease progression [15].

Therefore, the current multicenter study aimed to compare hospitalized COVID-19 patients treated with MOL with those who did not receive antiviral therapy to determine the potential clinical benefit, filling this knowledge gap. It is important to note that both our analysis and the study performed by Carlos et al. included patients hospitalized during the pandemic wave of the Omicron variant [15], whereas recruitment to the MOVe-IN study occurred during a period of the predominance of previous SARS-CoV-2 variants of concern, Alpha and Delta [8].

Despite the altered sensitivity of the Omicron mutation to the vaccines and some COVID-19 therapeutics, data available from in vitro studies indicate that the antiviral drugs, remdesivir, nirmatrelvir/ritonavir, and MOL remain active against this variant of concern [2]. The study population consisted of patients requiring hospitalization due to COVID-19 and burdened with risk factors of severe disease course related to comorbidities and age; 73% were patients over 60 years of age, whereas in the MOVe-IN study, such patients accounted for only 42% [8]. Also noteworthy is the significantly higher proportion of women in our analyzed group compared to the MOVe-IN study (56% vs. 43%), which may have influenced the result.

The present analysis demonstrated that the use of MOL was associated with a significant reduction in all-cause mortality compared to using no antiviral drugs, 9.9% vs. 16.3% (*p = *0.03). Thus, our data are consistent with the results obtained by Carlos et al., who reported a mortality rate in the MOL-treated group of 8.9% versus 15.9% in the control group (*p < *0.001) [15]. In our study, the difference was most pronounced for the subset of patients aged over 80 years with baseline oxygen saturation ≤ 95% who received MOL within the first 5 days from the onset of symptoms, 14.6% vs. 35.2% (*p = *0.016). Due to the fact that the baseline indices of the disease activity did not differ significantly in the two groups, the higher frequency of immunomodulating drugs among patients not treated with antivirals should be considered as reflecting the progression of the disease later in the course of the disease.

We found no statistically significant differences in terms of the need for mechanical ventilation, in contrast to a report from Hong Kong, which showed a significantly lower risk of this event in patients treated with MOL compared to the group not treated with antivirals, 0.4% vs. 1.4% (*p < *0.001) [15]. These discrepancies in the results may have been influenced by the difference in the size of the cohorts analyzed, 203 vs. 2116 patients. It is noteworthy, however, that in our study none of the 41 patients older than 80 years with baseline oxygen saturation ≤ 95% who began MOL therapy within the first 5 days required mechanical ventilation, contrary to 3 out of 91 patients in the group not receiving antiviral therapy. The finding of a statistical association between the timing of MOL inclusion and its efficacy in the MOVe-OUT and MOVe-IN trials became the basis for recommending its use up to five days after the onset of disease symptoms [7–10].

In the population of patients over 80 years of age with baseline saturation ≤ 95% who received therapy within the first five days, the trend of more rapid clinical improvement as assessed by the WHO ordinal scale was also most evident, although this association was demonstrated in the entire group of patients receiving MOL compared with those not receiving antiviral treatment.

We are aware that although our study provides a valuable addition to the knowledge of MOL use in patients with COVID-19, it has some limitations related to its real-world and multicenter retrospective observational nature with possible bias and entry errors. However, its real-world nature is also the strength of the study, as the analyzed population is heterogeneous, covering different parts of the country, which allows the results to be generalized. The study did not take into account the vaccination status or past SARS-CoV-2 infections. In the analyzed period, the majority of the Polish population underwent one of these forms of immunization, and the Omicron variant had distinct immunological characteristics from the earlier variants on the basis of which the vaccine was created and with which patients could have come into contact before. Therefore, we believe that the immune status acquired prior to 2022 could not have had a significant impact on the efficacy of an antiviral drug devoid of immunomodulatory activity.In conclusion, we have documented that the use of MOL during the first five days in patients hospitalized for COVID-19 and burdened with risk factors for severe disease significantly reduces mortality and contributes to clinical improvement. This relationship is particularly evident in patients over 80 years of age and baseline oxygen saturation ≤ 95%.

## Data Availability

The datasets generated during and/or analyzed during the current study are available from the corresponding author on reasonable request.

## Abbreviations

COVID-19: Coronavirus disease 2019
CRP: C-reactive protein
ECMO: Extracorporeal membrane oxygenation
FDA: Food and Drug Administration
NO AVT: No antiviral therapy
IL-6: Interleukin 6
MOL: Molnupiravir
PTEiLChZ: Polish Association of Epidemiologists and Infectiologists
RT-PCR: Real-time reverse transcriptase–polymerase chain reaction
SARS-CoV-2: Severe acute respiratory syndrome coronavirus 2
SpO_2_: Oxygen saturation
WBC: White blood cells
WHO: World Health Organization
